# Breastfeeding of Aboriginal and/or Torres Strait Islander infants from a small rural cohort in Australia

**DOI:** 10.1186/s13006-025-00708-6

**Published:** 2025-03-28

**Authors:** Oyepeju M. Onifade, Saije K. Endacott, Tracy Schumacher, Kym M. Rae, Kirsty G. Pringle, the Gomeroi Gaaynggal Advisory Committee

**Affiliations:** 1 School of Medicine and Public Health, University of Newcastle, Callaghan (Awabakal Country), NSW Australia; 2 Women’s Health Research Program, Hunter Medical Research Institute, New Lambton Heights (Awabakal Country), NSW Australia; 3 School of Biomedical Science and Pharmacy, University of Newcastle, Callaghan (Awabakal Country), NSW Australia; 4 Food and Nutrition Program, Hunter Medical Research Institute, New Lambton Heights (Awabakal Country), NSW Australia; 5 Department of Rural Health, University of Newcastle, Tamworth (Gomeroi/Kamilaroi Country), NSW Australia; 6 Mater Research Institute, Aubigny Place, South Brisbane (Turrbal and Yuggera Country), QLD Australia; 7 Faculty of Medicine, University of Queensland, Herston (Turrbal and Yuggera Country), QLD Australia

**Keywords:** Breastfeeding, Childhood nutrition, First Nations, Infant feeding

## Abstract

**Background:**

Exclusive breastfeeding is recommended during an infant’s first six months of life as it is the optimal way to meet the infants nutritional needs. The aim of this study was to describe the breastfeeding intentions and practices of mothers carrying First Nations babies in the Gomeroi Gaaynggal longitudinal cohort.

**Methods:**

This study uses a subset of data from participants recruited between 2010–2018. Mothers carrying First Nations babies were recruited to the study at any stage during pregnancy (*N* = 425) at the Tamworth Rural Referral Hospital by First Nations research assistants. Breastfeeding intentions and previous pregnancy history data were obtained from participant survey and/or hospital antenatal records (*n* = 246). Infant breastfeeding details were obtained from mothers who agreed to participate in the follow-up study (*n* = 131/182) using participant surveys at approximately 3-, 6-, 9-, 12- and 24-months.

**Results:**

Most of the mothers (72.8%; 179/246) indicated an intention to breastfeed their infants exclusively after birth. Most infants (83.9%; 104/124) received some form of breast milk (either directly from the breast or as expressed breast milk). The median breastfeeding duration of infants in this study was 35 days/5 weeks (IQR: 14–150 days/2–21.4 weeks). 35.8% (19/53) of mothers reported having trouble with breastfeeding.

**Conclusion:**

Findings from this study show that breastfeeding initiation rates are similar to those reported for First Nations people living in non-remote areas of Australia. Further investigations are required to identify factors contributing to the short breastfeeding duration observed in this cohort.

## Background

Breastfeeding is an important component of early childhood nutrition with short- and long-term health benefits for both infants and their mothers [1, 2]. Breast milk is well-suited to exclusively meet infants’ nutrient requirements for adequate growth in the first six months [3]. The numerous bioactive molecules contained within breast milk including growth factors, prebiotics, anti-infectious, and anti-inflammatory agents protect against infection and inflammation and contribute to organ development, immune maturation and healthy microbial colonisation [4]. The nutritional components and non-nutritive bio-active factors in breast milk make it superior to infant formula [4]. The World Health Organization (WHO) recommends initiating breastfeeding within an hour of birth, breastfeeding exclusively for the first six months of life and continuing breastfeeding up to two years of age and beyond [5].

Globally, breastfeeding rates are insufficient to protect women’s and children’s health [6]. The World Health Assembly Global Nutrition Targets 2025 aims to increase the rate of exclusive breastfeeding in the first six months to at least 50% [5]. In 2013–2018, rates of initiation of breastfeeding within one hour of birth and exclusive breastfeeding for infants less than six months globally were low (43% and 41%, respectively) [6]. Furthermore, although 70% of women worldwide continue to breastfeed their child until one year of age with food supplementation, breastfeeding rates decline to 45% by two years of age [6]. A single centre prospective study based in Sydney, Australia showed that 92% of women initiated breastfeeding in hospital [7]. Furthermore, between 2017–2018, 61% of Australian children were exclusively breastfed until four months [8].

Breastfeeding initiation rates among Aboriginal and Torres Strait Islander people (hereafter respectfully referred to as First Nations) are also high. The 2010 National Infant Feeding Survey reports that 94.9% of First Nations women in Australia initiated breastfeeding (indicated by the number of children ever breastfed) and 61.4% of First Nations children were receiving breast milk at two months [9]. Similar initiation rates have been reported in First Nations cohorts in South Australia and Victoria, which reported initiation rates of 86% and 87.2%, respectively [10, 11]. Additionally, in the South Australian cohort, the number of infants receiving any breast milk declined to 54% by three months [10], reflecting the decline in breastfeeding rates seen nationally. Prior to colonisation, First Nations mothers exclusively breastfed their infants for at least six months and continued up until four years of age [12]. It has also been reported that children were generally breastfed until the arrival of a new sibling [13]. However, colonisation disrupted traditional breastfeeding practices with the separation of children from their mothers, removal of First Nations girls from their communities for domestic service, and rapid urbanisation [12, 14, 15]. This prohibited the handing down of traditional breastfeeding practices in many communities and women were often deprived of adequate guidance on breastfeeding from close family and community members [14].

The Gomeroi Gaaynggal Study is a longitudinal cohort study that follows women carrying First Nations babies from pregnancy into early childhood. The study is based in regional New South Wales (NSW) and aims to investigate the developmental origins of health and disease among First Nations Australians and develop programs with the community to strengthen health and well-being outcomes during and after pregnancy. Preliminary data showed that breastfeeding initiation rates among women in this cohort were 85.9%, however, there was initially limited evidence available on breastfeeding patterns [16]. Now that the study has been completed, we aimed to describe the breastfeeding intentions and practices of participants in the Gomeroi Gaaynggal cohort.

## Methods

### Ethical considerations

Ethical approval for the study was obtained from the Hunter New England Research Ethics Committee (HNEHREC 08/05/21/4.01), the Aboriginal Health and Medical Research Council (654/08), the New South Wales Human Research Ethics Committee (HREC/08/HNE/129) and the University of Newcastle Human Research Ethics Committee (UONHREC No. H-2009–0177). The study was also supported by the Gomeroi Gaaynggal Advisory Committee, which includes community Elders, First Nations women, and members of local Aboriginal Community Controlled Organisations. The Gomeroi Gaaynggal Advisory Committee provided significant cultural knowledge to guide the research study, contributed to the design of the study and the interpretation of the data, as well as reviewing and approving this publication prior to submission for peer review.

### Study setting and design

The Gomeroi Gaaynggal Study is a prospective longitudinal cohort of mother–child dyads, conducted among First Nations people. The study is located in Tamworth, New South Wales (NSW), Australia and ran from 2010–2018. The study followed women throughout pregnancy and continued until early childhood, investigating the developmental origins of health and disease. Further details of the Gomeroi Gaaynggal Study have been reported elsewhere [16]. The present study uses a subset of data from the larger Gomeroi Gaaynggal Study.

### Recruitment

Participants were recruited by First Nations research assistants at antenatal services of the Tamworth Rural Referral Hospital. Women were recruited into the pregnancy study at any stage during pregnancy and were eligible to participate if they self-identified as Aboriginal and/or Torres Strait Islander, or they identified that the father of the infant identified as either or both (*N* = 425). The purpose of the study was explained and written informed consent to participate in the pregnancy study was obtained. Study visits during pregnancy occurred once per trimester, where participants met with First Nations research assistants at the Gomeroi Gaaynggal Centre and completed demographics surveys.

When participants were in their third trimester, First Nations research assistants asked women participating in the pregnancy study if they would like to participate in the follow-up study. If interest was expressed, written informed consent was obtained (*N* = 182). During the follow-up study participants attended up to four visits in the infant’s first year (at 3-, 6-, 9- and 12-months), and one visit during each subsequent year, making it a total of five visits in the infant’s first two years. During these visits, Infant Feeding Recall (IFR) data was collected (as described below).

### Data collection

**Fig. 1:**
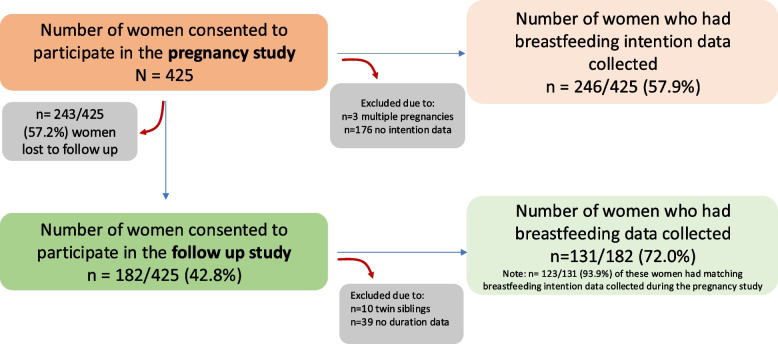
Participant flow through this study. There were 425 women recruited into the Gomeroi Gaaynggal pregnancy study. Of these, 246 women had feeding intention data collected. 182 women who had participated in the pregnancy study consented to participate in the Gomeroi Gaaynggal follow-up study. Of the 182 women who participated in the follow-up study, 131 had breastfeeding data collected. 123/131 women who participated in the follow-up study also had matching breastfeeding intention data. Women who had a multiple pregnancy were excluded from this study

All participants in the pregnancy study, who had a singleton pregnancy and had feeding intention data collected were included in this analysis (*n* = 246/425). Additionally, all participants who consented to the follow-up study, had a singleton pregnancy, and had breastfeeding data collected were included in the study (*n* = 131/182). It is important to note that 123/131 participants who were included in the follow-up also had matching feeding intention data collected during pregnancy. See Fig. 1 for more detailed information on participant flow through the study.

### Feeding intentions and obstetric data

Details about maternal demographics and pregnancy history (excluding First Nations status and maternal age) were obtained from hospital antenatal records and supplemented with data collected via participant surveys when data was not available in the hospital records. Information on First Nation status and maternal age were collected directly from participants upon recruitment and supplemented with information from hospital records. Data on feeding intentions were solely obtained from hospital antenatal records, where participants were asked by health professionals “What is the intention for feeding the baby this pregnancy?”. Based on the participants answer to this question health professionals picked from one of the following multiple choice options: “Breastfeeding, Expressed breast milk, Mixed, Formula, Undecided or Not Applicable”. For the purposes of this study “breastfeeding and expressed breast milk” were combined into one variable “intended to breastfeed exclusively”.

### Breastfeeding data

Details on the infant’s feeding practices were collected by a qualified dietitian or trained First Nations research assistant using questions from the Infant Feeding Recall (IFR) questionnaire, with the attending parent/guardian reporting on behalf of the infant. The IFR questionnaire collected information on the feeding practices of the infant in the child’s first year and at 24 months (as described above). It obtained details about breastfeeding initiation and duration, since the baby was born. It also asked mothers if they had trouble breastfeeding and what might have helped them continue. Questions included in the IFR were devised from the NSW Child Health Survey 2001 [17] and the 1995 National Nutrition Survey [18]. In this study breastfeeding was defined as any form of breastfeeding (direct from the breast or expressed) and included the duration of any breastfeeding (exclusive or not exclusive).

### Data analysis

Descriptive data analyses were performed using Stata/BE 17.0 (Stata Corp., College Station, TX, USA). As the IFR was collected multiple times throughout the follow-up study this presented a challenge when undergoing data analysis and decisions had to be made to best report the data collected. For data regarding whether the child was ever breastfed or breastfed after discharge from hospital, only the participant’s first answer to these questions was analysed, irrespective of further responses provided. For data regarding trouble breastfeeding, all responses regarding difficulties with breastfeeding were included. Breastfeeding duration was captured at the first instance participants self-reported that breastfeeding had ceased and did not include participants who reported that they were still breastfeeding at their last study visit (*N* = 79/131). The duration provided was self-reported as the total time their child had been breastfed, including weaning. Breastfeeding duration data were plotted on a survival curve in STATA, whereby the analysis time was breastfeeding duration, and the failure event was cessation of breastfeeding. For reporting purposes, one month was equal to 30 days, and one week was equal to 7 days.

## Results

Details regarding pregnancy history and feeding intentions were obtained for 246/425 mothers during pregnancy. Breastfeeding data was obtained from 131/182 mothers during the follow-up study (see Fig. 1). There was a high rate of unanswered pregnancy history and feeding intention data (42.1%; 179/425), highlighting a significant shortfall in that numerous participants are not being asked these questions by hospital staff or the answers are not being recorded sufficiently in their medical records. Additionally, there was a significant number of missing responses from the Infant Feeding Recall questionnaires completed in the follow-up study (281 out of a possible 665 entries were recorded), this is likely because not all women attended all visits.

Pregnancy history and demographic data are presented in Table 1. Briefly, most mothers identified as being First Nations (82.1%; 202/246). Of these, 34.5% (85/246) of participants identified that both themselves and their child’s father identified as First Nations while 47.6% (117/246) reported that their child’s father either did not identify or their status was unknown. Alternatively, 17.9% (44/246) of mothers did not identify as First Nations or their status was unknown but identified that the father of their baby was First Nations. The median age of participants in this study was 24.2 years, with most participants (76.8%; 189/246) aged between 20.0–34.9 years of age. Most women (67.3%; 136/202) did not smoke during pregnancy. Less than half of the participants were either a first-time (17.5%; 31/177) or second-time mother (28.2%; 50/177). Breastfeeding history data were available for 61 women. Of these, 36.1% (22/61) of women stated they had previously breastfed. Most (88.6%; 218/246) women delivered at term (37.0–41.9 weeks), 11% (27/246) delivered preterm (< 37 weeks) and 0.4% (1/246) delivered post-term (≥ 42 weeks). Most women gave birth vaginally (68.8%; 154/224), while 31.3% (70/224) gave birth via caesarean section. Most babies born in this study were male (56.1%; 108/246). Details of infant admissions to a Special Care Nursery or a Neonatal Intensive Care Unit after birth were available in the maternal medical records of 183 women. Of these, 24.6% of infants were reported to have spent time in a Special Care Nursery/Neonatal Intensive Care Unit after birth.

**Table 1:** Maternal demographics

Pregnancy history (*n* = 246)	Data available (n (%))	Median (IQR)	n (%)
First Nations status	246 (100)		
First Nations Mother		N/A	117 (47.6)
First Nations Father		N/A	44 (17.9)
Both First Nations		N/A	85 (34.5)
Maternal age (years)	246 (100)	24.2 (21.2–29.0)	
< 20			39 (15.9)
20.0–34.9			189 (76.8)
> 35			18 (7.3)
Smoked during pregnancy	202 (82.1)		
No		N/A	136 (67.3)
Parity	177 (72.0)	2 (1–3)	
First time mother			31 (17.5)
Second time mother			50 (28.3)
Third time mother or more			96 (54.2)
Breastfed a previous child?	61 (24.8)		
Yes		N/A	22 (36.1)
Gestational age at birth (weeks)	246 (100)	39.05 (38.1–40.1)	
Preterm (< 37)		36 (35.1–36.4)	27 (11.0)
Term (37.0–41.9)		39.2 (38.3–40.2)	218 (88.6)
Post term (≥ 42)		43 (N/A)	1 (0.4)
Mode of birth	224 (91.1)	N/A	
Vaginal			154 (68.8)
Caesarean Section			70 (31.3)
Infant sex	246(100)	N/A	
Male			138 (56.1)
Female			108 (43.9)
Baby spent time in NICU or Special Care Nursery after birth			
Yes	183 (74.4)	N/A	45 (24.6)

*IQR* interquartile range, *NICU* Neonatal Intensive Care Unit

Details of breastfeeding intention and practices are presented in Table 2. Most mothers (72.8%; 179/246) indicated an intention to breastfeed their infants exclusively after birth. Most infants (81.5%; 101/124) were breastfed after arrival at home from the hospital and 83.9% (104/124) of the infants were breastfed at some stage in infancy. A large percentage (35.8%; 19/53) of mothers who responded reported they had trouble breastfeeding their infants. Of the women who reported having trouble breastfeeding, when asked what might have helped them with continuing breastfeeding, 26.3% (5/19) mentioned latching support would have been helpful, 5.3% (1/19) identified the need for support from a lactation consultant. Help with other issues including milk production (21.1%; 4/19), cracked nipples (5.3%; 1/19), and physical breast issues (breasts being too big/small to breastfeed; 10.5%; 2/19) were also indicated.

**Table 2:** Breastfeeding intentions and characteristics

Breastfeeding intentions after birth (*n* = 246)	Data available (n (%))	n (%)
	246 (100)	
Intended to breastfeed exclusively		179 (72.8)
Intended to combine breastfeeding and infant formula		8 (3.2)
Intended to use infant formula only		40 (16.3)
Undecided		18 (7.3)
Not applicable		1 (0.4)
**Breastfeeding data (*n* = 131)**		
Child breastfed after discharge from hospital	124 (94.7)	
Yes		101 (81.5)
Child ever breastfed	124 (94.7)	
Yes		104 (83.9)
Had trouble breastfeeding	53 (40.5)	
Yes		19 (35.8)
No		34 (64.2)

**Fig. 2:**
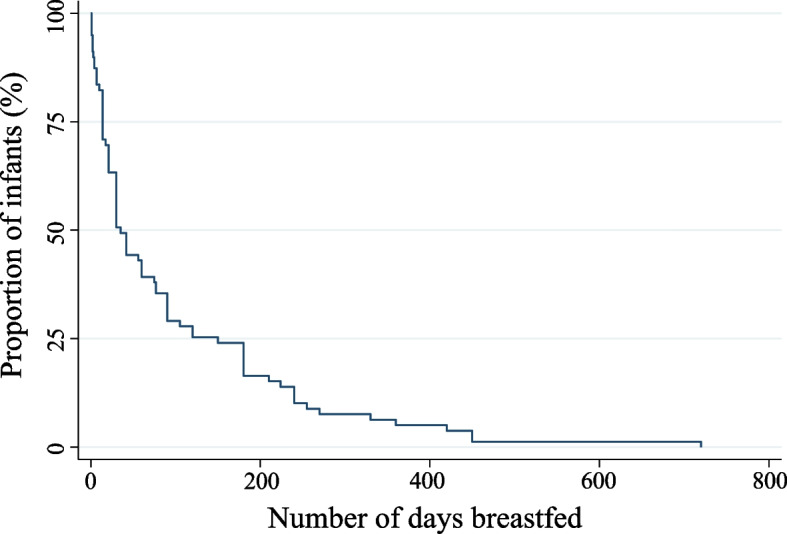
Breastfeeding duration of infants in the Gomeroi Gaaynggal cohort. Breastfeeding duration of infants in the cohort (*n* = 79)

Amongst those known to have stopped breastfeeding (79/131) the median duration of any form of breastfeeding was 35 days (IQR: 14–150; *n* = 79; Fig. 2). Of the infants who were breastfed, 12.7% (10/79) were breastfed for less than 1 week, 36.7% (29/79) were breastfed for between 7–30 days (~ 1 month), 25.3% (20/79) for 31–120 days (~ 1–4 months), 8.9% (7/79) were breastfed between 121–180 days (4–6 months), 11.4% (9/79) for 121–180 days (~ 6–12 months), and 5.1% (4/79) of the infants were breastfed beyond 12 months (Fig. 2). Responses regarding breastfeeding duration were unavailable for 39.7% (52/131) of the mothers. This was likely a result of women no longer coming to their follow-up appointments or having indicated that they were still breastfeeding at their 24-month appointment (*n* = 3). If women were ‘lost to follow-up’ we did not know when they stopped breastfeeding and hence could not determine their breastfeeding duration.

## Discussion

This study shows that breastfeeding initiation rates are high among First Nations infants in this cohort. However, breastfeeding duration declined quickly with the median duration of any form of breastfeeding (mixed or exclusive) being 35 days. Although most of the mothers planned to exclusively breastfeed their infants after birth, 70.9% had ceased breastfeeding by 3 months, indicating there might be several underlying factors influencing breastfeeding practices.

The social determinants of health greatly influence health inequities including breastfeeding outcomes [19]. While lower than those reported for First Nations women in the 2010 National Infant Feeding Survey (94.9%) [9], the rate of breastfeeding initiation in our study (83.9%; indicated by the number of infants that received mixed/exclusive breastfeeding) is consistent with findings from other studies in regional areas. A study involving First Nations women living in regional areas of South Australia reported breastfeeding initiation rates of 89% [10]. Our study also reported higher than or similar initiation rates than that reported for First Nations women living in major Australian cities (Melbourne, Sydney, and Brisbane; 84.5%, 67.4%, 59.0%, respectively) [20–22]. It is well-documented that breastfeeding rates decline after hospital discharge [23, 24]. The 2010 National Infant Health Survey reports that by two months 61.4% of First Nations children are receiving any amount of breast milk [9]. Similarly, this study shows that while 81.5% of the infants were breastfed at discharge from the hospital after birth, at 60 days (two months) after birth, 60.8% of infants were still receiving any breast milk (Fig. 2). Breastfeeding duration for First Nations women is affected by geographic location. Previous studies of breastfeeding duration among First Nations women in South and Western Australia, respectively, indicate that breastfeeding duration increases based on remoteness [10, 25] or living outside of major cities [10]. Mothers in the Gomeroi Gaaynggal cohort lived in a regional NSW city. Hence our data indicates a higher level of breastfeeding cessation in our study than those reported for First Nations populations living in both non-isolated areas of Western Australia (where 40.6% of infants were breastfed for less than 3 months or never breastfed) [25] and regional South Australian communities (where 41.4% of infants were breastfed for less than 12 weeks or never breastfed) [10]. Therefore, women in the Gomeroi Gaaynggal Study showed breastfeeding initiation rates similar to those reported for First Nations women living in regional areas, however, breastfeeding duration in the cohort is lower.

Cultural and historical factors strongly influence breastfeeding initiation and duration. Studies have shown that First Nations women seek help and support from people they trust such as their mothers, grandmothers and aunties [14]. Intergenerational transfer of knowledge has a significant impact on breastfeeding practices however, this can only be beneficial if the quality of information being shared is adequate and without disruption in the means of transfer [14]. First Nations lives have been impacted by colonisation and discriminatory societal attitudes resulting in a large disruption in sharing of information between generations [12, 14]. These disruptions have contributed to a negative breastfeeding experience extending through generations of First Nations women to date [14].

Although this study did not investigate associations between factors influencing breastfeeding practices and breastfeeding duration and initiation, previous studies may provide some insight on these. A study on factors associated with the initiation of breastfeeding by First Nations mothers in Perth has shown that location, maternal education, partner’s perceived feeding method preference, parity, maternal age and grandmother influence could affect breastfeeding practices [23]. Binns et al*.* showed a strong association between initiation and partner perception as mothers were six times more likely to breastfeed at discharge if the infant’s father preferred breastfeeding [23]. Similarly, a study investigating maternal perception of partner support during breastfeeding in the USA also found that women who experienced positive and active partner support had an increased confidence in their ability to breastfeed compared to women who did not [26]. A recent systematic review of the factors associated with breastfeeding initiation and maintenance, identified that living remotely, attending a First Nations specific service, higher education (≥ year 10) and maternal age (> 25 years), living in larger households (≥ 4 people), and having a partner, as protective factors associated with breastfeeding in First Nations Australian women [27]. The need for culturally safe maternity care is a national priority and multiple studies have demonstrated the success of such models of care on breastfeeding outcomes. A prospective, non-randomised interventional trial based in Brisbane, Australia, showed that the implementation of a Birthing in our Community service resulted in an increased likelihood that First Nations women would exclusively breastfeed on discharge from hospital [28]. This is supported by data from a Melbourne cohort where the introduction of a First Nations-specific caseload midwifery model demonstrated rates of breastfeeding initiation (96%) and duration well above those reported nationally for First Nations Australians (reported above) [9], with 71% of women providing breast milk at 3 months [29].

Another major contributory factor to breastfeeding outcomes, particularly in relation to duration, is difficulty in breastfeeding. This study reports that 35.8% (*n* = 19/53) of mothers who responded, had trouble breastfeeding. When asked what may have helped them with continuing breastfeeding, support from a lactation consultant as well as support with latching, cracked nipples, milk production, thin breast milk/reflux and physical breast issues were reported as factors that would have facilitated breastfeeding continuance. Adequate lactation support may improve breastfeeding outcomes, especially in primiparous women. The shortage of, and duplication or multiplication of tasks by lactation consultants in rural hospitals may also contribute to this as there are limitations to the amount of time spent with the mothers by the staff [30, 31]. With regards to breastfeeding, women are less likely to discuss their experiences with people they do not have any relationship with and therefore do not trust [31]. A First Nations lactation consultant may improve the likelihood of First Nations women receiving breastfeeding support that is culturally safe and appropriate.

The proportion of first-time mothers (17.5%) and admissions to NICU or Special Care Nursery (24.6%), may have also influenced the breastfeeding rate observed (Table 1). Studies have shown that women are more likely to breastfeed their baby if they have breastfed previous babies [23]. Less than half of the respondents in this study reported breastfeeding previous babies, which could have contributed to the low breastfeeding duration in this cohort. Additionally, it is known that infants who are admitted to the NICU experience lower breastfeeding rates than those who are not admitted [32]. This has been associated with factors such as prematurity, delayed initiation of direct breastfeeding, maternal stress, lower rates of skin-to-skin contact and maternal-infant bonding and longer hospital stays, in NICU admitted infants [32]. This cohort had a preterm birth rate of 11% and approximately one quarter of infants in this study experienced a NICU or Special Care Nursery admission, both of which could have significantly impacted breastfeeding duration in this study.

The limitations emerging in this study mean that the findings should be interpreted with caution. The sample size is small, and this study was conducted in a regional community of New South Wales and while the findings may be useful in other communities, they are not necessarily generalisable to the larger First Nations population. The high rate of missing responses observed is a major limitation in this study. Several factors, including how and when data was collected, and social and cultural factors, could have contributed to the high rate of missing data. Importantly, there was a high rate of unanswered pregnancy history and breastfeeding intention data. These data were collected from the participants’ medical records, thereby indicating that either a significant number of participants are not being asked these questions by hospital staff or the answers are not being recorded sufficiently in their medical records. Inadequate collection of breastfeeding data in Australian state-based databases has been raised by others [33]. There is the likelihood of recall challenge with some of the responses to questions about when participants stopped breastfeeding due to the time between the event occurring and completing the survey. The responses to questions regarding feeding intentions may not be truly reflective of mothers’ intentions as the stigma attached to not breastfeeding is extremely high and many mothers do not want to be perceived as not doing what is right for their babies [34, 35]. It may be that First Nations women feel pressured to provide certain answers, particularly in hospital settings [14].

Investigating breastfeeding practices and factors influencing such practices among First Nations populations is a body of work that requires people trusted by the community to undertake. While findings from this study will help the community to understand areas where intervention may be needed, further investigations are required to identify factors that can support families to ensure infants are breastfed for longer.

## Data Availability

The data presented in this study are available on request from the corresponding author and the Gomeroi Gaaynggal Advisory Committee. The data are not publicly available for ethical reasons.

## Abbreviations

HNEHREC: Hunter New England Health Research Ethics Committee
IFR: Infant Feeding Recall
NSW: New South Wales
WHO: World Health Organization
